# The risk of ischemic optic neuropathy post phacoemulsification cataract surgery

**DOI:** 10.11604/pamj.2017.28.53.10806

**Published:** 2017-09-21

**Authors:** Mousa Victor Al-Madani, Nancy Khalaf Al-Raqqad, Naser Abdallah Al-Fgarra, Amal Mousa Al-Thawaby, Ahmed Abdelra’of Jaafar

**Affiliations:** 1 King Hussein Medical Center, Royal Medical Services of Jordanian Armed Forces, Amman 11733, Jordan

**Keywords:** Optic neuropathy, non arteritic, ischemic and phacoemulsification

## Abstract

**Introduction:**

The aim was to study the risk of non arteritic ischemic optic neuropathy after phacoemulsification cataract surgery.

**Methods:**

This study was conducted at King Hussein Medical Center during the period between January 2015 and July 2016. Patients attending ophthalmology clinic complaining of decreased vision due to lens opacity were evaluated. Patients were divided into two groups. First group included patients with no medical illness and second group included patients with diabetes mellitus, hypertension or hyperlipidemia. The two groups were further divided into two subgroups. First subgroup included patients who had phacoemulsification surgery and second subgroup did not have surgery. All patients were followed up for 6 months. They were assessed by neuro-ophthalmologist looking for ischemic optic neuropathy.

**Results:**

A total number of 568 patients were enrolled. Group 1A included patients with no medical illness who underwent surgery and group 1B did not undergo surgery. The number of patients in these two subgroups was 119 and 103 respectively. Number of patients in group 2A (medical illness and surgery) was 188 and number of patients in group 2B (medical illness and no surgery) was 130. The incidence of ischemic optic neuropathy was 4.3 % in group 2A, 4.2 % in group 1A, 0.8% in group 2B, and 0% in group 1B.

**Conclusion:**

Phacoemulsification is a risk factor for non arteritic ischemic optic neuropathy independent of the presence of medical risk factors. Suggested mechanisms would be local anaesthesia, intraocular pressure fluctuation and local intraocular inflammation.

## Introduction

Ischemic optic neuropathy can be arteritic or non arteritic [1]. The non arteritic type is relatively common with estimated annual incidene of 2 to 10 per 100000 population aged more than 50 years [2, 3]. Medical illness such as diabetes mellitus, hypertension, hyperlipidemia and cigarette smoking are definite risk factors for its occurrence. The pathophysiology for its development is suggested to be ischemia in the posterior ciliary circulation affecting optic nerve blood flow [4]. The role of cataract surgery in developing ischemic optic neuropathy is not well established and has been an issue of controversy [5–10]. We excluded patients with glaucoma or with previous optic neuropathy in the ipsilateral eye as they have vulnerable discs or discs at risk. Patients were categorized into two groups in order to evaluate the risk of cataract surgery on development of ischemic optic neuropathy and whether it is dependent on presence of medical risk factors or not. The type of anaesthesia was also selected to be local for all patients. Patients who underwent surgery under general anaesthesia were excluded to rule out the effect of general anaesthesia in causing optic neuropathy. Patients with complicated surgeries such as posterior capsule rupture were also excluded as they may have prolonged intraocular inflammation.

## Methods

This study was conducted at King Hussein Medical Center during the period between January 2015 and July 2016. Patients attending ophthalmology clinic complaining of decreased vision due to lens opacity were evaluated. Patients were divided into two groups. First group (group 1) included patients with no medical illness and second group (group 2) included patients with diabetes mellitus, hypertension or hyperlipidemia. The two groups were further divided into two subgroups. First subgroup included patients who had phacoemulsification surgery and second subgroup did not have surgery. Reasons for not doing the surgery included not keen patients or patients listed on a waiting list exceeding 6 months. Exclusion criteria included presence of glaucoma, previous optic neuropathy in the ipsilateral eye, complicated surgeries or surgeries done under general anaesthesia. All patients were followed up for 6 months after their surgeries for those who underwent the surgery or for 6 months after their first presentation for those who did not have surgery. The surgical technique procedure was the same for all patients who underwent surgeries. Patients were assessed by neuro-ophthalmologist looking for ischemic optic neuropathy. Ocular examination included Snellen’s chart visual acuity, intraocular pressure measurement, anterior and posterior segment examination. When ischemic optic neuropathy were suspected, further tests were done including assessment of pupil, color vision, Humphrey’s 24-2 visual field testing. Fluorescein angiography and optical coherent tomography were done in selected patients when indicated. The onset of ischemic optic neuropathy whether it was early or late was also recorded. The presence of previous ischemic optic neuropathy in the contra lateral eye was recorded.

## Results

A total number of 568 patients were enrolled. Mean age was 62.4 years with a male to female ratio of 1.1 to 1. Twenty eight patients were excluded leaving 540 patients enrolled (Table 1). Causes of exclusion were glaucoma, general anaesthesia, complicated surgeries and ipsilateral optic neuropathy. The last category included two patients with previous ischemic optic neuropathy in the eye planned for surgery. Four of the six complicated surgeries had posterior capsule rupture and the remaining two patients had anterior capsular extension during capsulorrhexis and the surgery was converted to extra capsular cataract extraction.

**Table 1:** Excluded patients

Cause of exclusion	Number of patients
Glaucoma	13
Ipsilateral optic neuropathy	2
General anaesthesia	7
Complicated surgery	6
Total	28

Group 1A included patients with no medical illness who underwent surgery and group 1B did not undergo surgery. The number of patients in these two subgroups was 119 and 103 respectively. Number of patients in group 2A (medical illness and surgery) was 188 and number of patients in group 2B (medical illness and no surgery) was 130 (Table 2). A total number of 14 patients had ischemic optic neuropathy. The vast majority of them (13 out of 14) occurred in patients who had phacoemulsification surgery and only one occurred in group who did not undergo surgery. Among these 14 patients 9 had medical illnesses and 5 had no illness. Table 3 shows the distribution of medical illnesses.

**Table 2:** Distribution of patients

Group	Total number of patients		Patients with ischemic optic neuropathy		P-value
	Number	Percentage	Number	Percentage	
1A (no medical illness and surgery)	119	22%	5	0.9%	0.031
1B (no medical illness and no surgery)	103	19.1%	0	0	
2A (medical illness and surgery)	188	34.8%	8	1.5%	0.033
2B (medical illness and no surgery)	130	24.1%	1	0.2%	
Total	540	100%	14	2.6%	

**Table 3:** Distribution of medical illnesses

Category		Number of patients
No medical illness		222
Medical illness	Diabetes mellitus	74
	Hypertension	86
	Hyperlipidemia	49
	Combined	109

The incidence of ischemic optic neuropathy was 4.3 % in group 2A, 4.2 % in group 1A, 0.8% in group 2B, and 0% in group 1B (Table 4). The incidence of ischemic optic neuropathy in patients who underwent phacoemulsification was statistically significant regardless whether they have medical illness or not (4.3% and 4.2% versus 0.8% and 0%, p= 0.033 and p= 0.031).

**Table 4:** Distribution of ischemic optic neuropathy patients and statistical significance

Group	Number of patients	Number of ischemic optic neuropathy patients	Percentage within their subgroup	P-value
1A (no medical illness and surgery)	119	5	4.2%	0.31
1B (no medical illness and no surgery)	103	0	0	
2A (medical illness and surgery)	188	8	4.3%	0.33
2B (medical illness and no surgery)	130	1	0.8%	

The number of patients with contralteral ischemic optic neuropathy was 21. Four of the patients with ischemic optic neuropathy had a previous attack of ischemic optic neuropathy in the contralteral eye (p = 0.0008).

## Discussion

The relationship between ischemic optic neuropathy and cataract surgery is controversial [6–8]. There are reports that proved such relationship especially in patients who had previous attack of ischemic optic neuropathy in the contralteral eye [5, 7]. The exact mechanism is poorly understood. It is well known that the pathophysiology of ischemic optic neuropathy is ischemia in the posterior ciliary circulation affecting optic nerve blood flow which is not supposed to occur after uneventful phacoemulsification surgery.

Ischemic optic neuropathy after cataract surgery in some reports was divided into early and late types. A postulated mechanism for early type is indisputably elevated intraocular pressure [2]. Glaucoma and sustained increased intraocular pressure are causes of optic nerve insult [11, 12]. On the contrary, a transient intraocular pressure elevation role in causing optic neuropathy is doubtful.

We excluded patients with vulnerable discs from our study such as glaucoma and previous ipsilateral ischemic optic neuropathy (Table 1). A total number of 28 patients were excluded; almost half of them had glaucoma. All of our patients underwent surgery under retro bulbar local anaesthesia. None of the patients had retro bulbar hematoma that may affect the optic nerve. Patients with complicated surgery were also excluded as they may have a prolonged post operative inflammation.

A total number of 14 patients were found to have ischemic optic neuropathy in the first 6 months post operatively representing 2.6% of the 540 patients enrolled. Table 2 shows that the total number of patients with no medical illness (group 1) was 222 (41.1% of patients) compared to 318 patients with medical illness (group 2, 58.9%). Five of patients (0.9%) of group 1 had optic neuropathy compared to 9 patients (1.7%) of group 2. This means that the occurrence of optic neuropathy in the operated eye is not significantly related to the presence of medical illness (p=0.4). On the other hand the number of operated patients (group 1A + 2A) was 307 compared to 233 non operated patients (group 1B + 2B). Optic neuropathy occurred in 13 of the operated patients (2.4%) and in one non operated patient (0.2%). This is statistically significant with p =0.0008. If we study the risk in each group alone; the risk of occurrence of optic neuropathy in the operated eye in those with medical illness and those with no medical illness the result will be also statistically significant (p =0.033 and p=0.031, Table 4. The conclusion we made is that non arteritic ischemic optic neuropathy significantly occurred after uneventful phacoemulsification independent on the presence of medical risk factors.

Twenty one patients were found to have non arteritic ischemic optic neuropathy on the contralteral eye prior to undergoing surgery. Four of them (19%) developed ischemic optic neuropathy in the operated eye. This is statistically significant (p =0.0008) taking into consideration that the risk of developing ischemic optic neuropathy post surgery in our series was 2.6%. This figure is less than what was observed by Lam BL et al [5] where 53% of patients with contralteral ischemic optic neuropathy developed neuropathy in the operated eye.

Half of the 14 patients the insult occurred early in the first week after surgery. The most likely postulated theory to explain this is related to local anaesthesia injection. None of the patients had retro bulbar hematoma or extraordinary hemorrhage. Retro bulbar local anaesthesia was reported to cause optic neuropathy [13, 14]. Other postulated theory for this early damage is the transient fluctuation in intraocular pressure which was reported by Elston J [2]. The remaining 7 patients had late ischemic optic neuropathy occurring after one month post surgery. The most likely explanation would be a locally mediated inflammation due to vasoactive peptide release that may cause cystoid macular edema and possibly the progression of ischemic diabetic intraocular complications [2].

## Conclusion

Phacoemulsification cataract surgery even if uneventful is a risk factor for non arteritic ischemic optic neuropathy independent of the presence of medical risk factors. Patients with contralateral ischemic optic neuropathy are at higher risk for developing it. Suggested mechanisms would be local anaesthesia, intraocular pressure fluctuation and local intraocular inflammation.

### What is known about this topic

- Non arteritic ischemic optic neuropathy is due to ischemia;
- Phacoemulsification increases the risk of non arteritic ischemic optic neuropathy. This was not studied in relation with medical illnesses.

### What this study adds

- Phacoemulsification increases the risk of non arteritic ischemic optic neuropathy independent of the presence of medical risk factors;
- Patients with contralateral ischemic optic neuropathy are at higher risk for developing it.

## Competing interests

The authors declare no competing interests.
